# The importance of external validation to advance precision psychiatry

**DOI:** 10.1016/j.lanepe.2022.100498

**Published:** 2022-08-26

**Authors:** Dominic Oliver

**Affiliations:** Early Psychosis: Interventions and Clinical-detection (EPIC) Lab, Department of Psychosis Studies, Institute of Psychiatry, Psychology & Neuroscience, King's College London, London, UK

Patients with a psychotic disorder are more likely to develop a cardiometabolic disorder compared to the general population, resulting in a reduction in life expectancy of around 15 years.^1^ Precision psychiatry tools use individual-level patient data in clinical prediction models to stratify risk and inform clinical decision making. Precision psychiatry tools can therefore improve early identification of people at higher risk of poor cardiometabolic outcomes, help tailor therapeutic strategies and improve prevention of cardiometabolic diseases long-term. While clinical prediction models are routinely used to monitor cardiometabolic outcomes, the Psychosis Metabolic Risk Calculator (PsyMetRiC) was the first to be developed to perform this role in young people with psychosis (aged 16–35; *n* = 651) before being externally validated in the UK (*n* = 510).^2^ PsyMetRiC uses age, Black/African Caribbean ethnicity, Asian/Other ethnicity, sex, body mass index, current smoking status, prescription of a metabolically-active antipsychotic and high-density lipoprotein and triglyceride concentrations to predict risk of metabolic syndrome up to 6 years. Perry and colleagues’ paper^3^ in this issue of Lancet Regional Health - Europe presents results from two further external validations in Spain (*n* = 466) and Switzerland (*n* = 558), showing that PsyMetRiC retains its predictive performance. These results suggest that PsyMetRiC is viable for implementation in clinical sites across Western Europe to improve early identification of cardiometabolic risk and inform personalised treatment decisions for young people with psychosis.

Patients with severe mental illness have an increased risk of cardiovascular disease morbidity and mortality.^4^ In the case of psychosis in particular, negative cardiometabolic outcomes are exacerbated by antipsychotic treatment,^5^ meaning that appropriate, evidence-based mental healthcare can reduce severity of presenting psychotic symptoms at the cost of negatively impacting physical health. Psychiatry has progressed towards prioritisation of preventive approaches and early intervention, and it is evident that a holistic approach, encompassing both mental and physical health, is essential to improve long-term outcomes. Tools like PsyMetRiC can be very valuable in informing clinical decisions to positively influence physical health in psychiatric populations, in this case by identifying patients who may benefit from prescription of a less metabolically-active antipsychotic.

The utility of precision psychiatry tools is ultimately dependent on the quality, relevance and generalisability of the data on which it is developed. However, generalisability of precision psychiatry tools is currently poor. Meta-analytical evidence has shown that while over 600 individualised clinical prediction models have been published in psychiatry, only 5% have been externally validated (performance tested in an independent sample) and less than 1% tested for implementation.^6^

For precision psychiatry to meaningful impact real-world clinical care, more research teams need to similarly prioritise robust external, international validation studies to test performance in new settings with corresponding differences in patient sociodemographics and service configuration. Advances in this area are reliant on fostering collaborations between research teams, particularly internationally, to allow for model performance to be tested on varied, representative and information governance-compliant datasets. A model that is resistant to sample differences is suited to implementation in a variety of settings and resistant to changes in population characteristics over time.^7^ Ensuring this robustness is a key ethical consideration as insufficiently generalisable models often lead to disproportionately high error rates in marginalised and vulnerable populations (e.g. ethnic minorities).^8^

While PyMetRiC performs well in Western European settings, future work should investigate its performance in other non-European and more ethnically/racially diverse settings. This is particularly important due to ethnic and racial differences in cardiovascular risk^9^ that may currently be under-represented in prior datasets. The current performance of the model is considered to be acceptable but could be further improved through refinement of existing predictors (e.g. more detailed representation of ethnicity), addition of new predictors (e.g. comorbid diagnoses) or more complex statistical techniques (e.g. supervised machine learning), either alone or combined. However, it is worth noting that more complex modelling methodologies may also have a negative impact on interpretability and generalisability, therefore caution is needed.

Barriers and facilitators for future implementation of PsyMetRiC are also important to consider. Currently, the evidence suggests that the most important facilitators for effective implementation involve employing appropriately-skilled staff and optimising service configuration.^10^ Moreover, the current model is reliant on individuals having complete data, which may not be viable for prospective use. Delaying predictions until all predictor data is collected may not be feasible, but similarly, making predictions early before essential predictor information is available will result in inaccurate predictions. Data may also be missing for people less able to attend clinical visits, potentially disproportionately impacting people who are more unwell without requiring inpatient care. Ethical and practical considerations need to be made when progressing PsyMetRiC (and other similar models) to prospective, clinical use.

Precision psychiatry is a quickly advancing field. To make a positive and equitable impact on real-world mental healthcare, more external validation studies and considerations for prospective clinical use need to be published.

## Declaration of interests

None to declare.
